# Dynamic species interactions associated with the range-shifting marine gastropod *Mexacanthina lugubris*

**DOI:** 10.1007/s00442-022-05128-5

**Published:** 2022-03-07

**Authors:** Piper D. Wallingford, Cascade J. B. Sorte

**Affiliations:** 1 Department of Ecology and Evolutionary Biology, University of California, Irvine, CA USA; 2 Department of Ecology and Evolutionary Biology, University of California, Los Angeles, CA USA

**Keywords:** Range shifts, Climate change, Community ecology, Species interactions, Rocky intertidal

## Abstract

Globally, species are undergoing range shifts in response to climate change. However, the potential impacts of climate-driven range shifts are not well understood. In southern California, the predatory whelk *Mexacanthina lugubris* has undergone a northward range shift of more than 100 km in the past four decades. We traced the history of the whelk’s range shift and assessed potential effects using an integrated approach, consisting of field surveys, as well as feeding and thermotolerance experiments. We found that at sites where *Mexacanthina* and native species co-occurred, native whelks distributions peaked lower in the intertidal. In laboratory experiments, we found that the presence of *Mexacanthina* led to reduced growth in native whelks (*Acanthinucella spirata*). Additionally, the range-shifting whelk was able to tolerate higher temperatures than common native species (*A. spirata* and *Nucella emarginata*), suggesting further impacts as a result of climate warming. Many species are likely to undergo range shifts as a coping mechanism for changing climatic conditions. However, communities are unlikely to shift as a whole due to species-specific responses. By studying the impacts of range-shifting species, like *Mexacanthina*, we can better understand how climate change will alter existing community structure and composition.

**Supplementary Information:**

The online version contains supplementary material available at 10.1007/s00442-022-05128-5.

## Introduction

Climate change is altering populations and communities at an unprecedented scale, with the potential for irreversible losses of biodiversity (Bellard et al. 2012). As climate change continues and accelerates, many species are expected to become extinct, either locally or globally (Root et al. 2003; Thomas et al. 2004). Range size can be an important predictor of extinction, with highly localized species at the greatest risk (Brooks et al. 2002; Sekercioglu et al. 2008). Range shifts can, therefore, present an opportunity for persistence as populations shift to more hospitable climates, limiting losses and protecting global diversity. (Chen et al. 2011; Dawson et al. 2011). Climate-induced range shifts can occur at a variety of scales, including latitudinal shifts, as well as changes in elevation or depth (Parmesan and Yohe 2003; Root et al. 2003; Chen et al. 2011), and have been reported across taxa and ecosystems (Parmesan 2006; Sorte et al. 2010; Poloczanska et al. 2013).

The impacts of novel species in communities have been well studied in the invasion literature, and range-shifting species may similarly alter community dynamics. Range shifts vary greatly in rate and extent (Chen et al. 2011), and communities are unlikely to shift as a whole in response to climate change. Asynchronous and heterogeneous species responses can result in altered species interactions similar to those seen in non-native species introductions and invasions (reviewed in Wallingford et al. 2020). However, few studies have assessed the effects of range-shifting species (e.g., species that are not directly introduced by anthropogenic activity) as they establish in new communities, despite the potential for significant impacts to communities and ecosystems (Sorte et al. 2010; Pecl et al. 2017; Aguilera et al. 2020).

Range-shifting species can consume, parasitize, or compete with native species that lack the ability or defenses to overcome them (Nackley et al. 2017). For example, a poleward range shift of the long-spined sea urchin *Centrostephanus rodgersii* has led to declines in abundance, fitness, and survival of blacklip abalone (*Haliotis rubra*) due to increased resource competition (Strain and Johnson 2009; Ling et al. 2009). Similar to invasive species, impacts are likely to be greatest when the non-native species is abundant or occupies a high trophic level (Bradley et al. 2019). Furthermore, rare communities or those that have already experienced disturbance may be more susceptible to negative impacts (Dale et al. 2001). Range shifts can lead to trophic mismatches (Tylianakis et al. 2010) and result in novel communities with no current analogs (Williams and Jackson 2007). However, there are important distinctions between range-shifting species and introduced species, namely that range-shifting species often share an evolutionary history with other species in its expanded territory (Sorte et al. 2010; HilleRisLambers et al. 2013). Overlap of species composition and interactions can be similar to those in the range-shifter’s original community, although naïve individuals in the new community can alter these dynamics (Wallingford et al. 2020) Understanding how communities will respond to range shifts is, therefore, an important consideration for developing conservation and management practices.

**Fig. 1:**
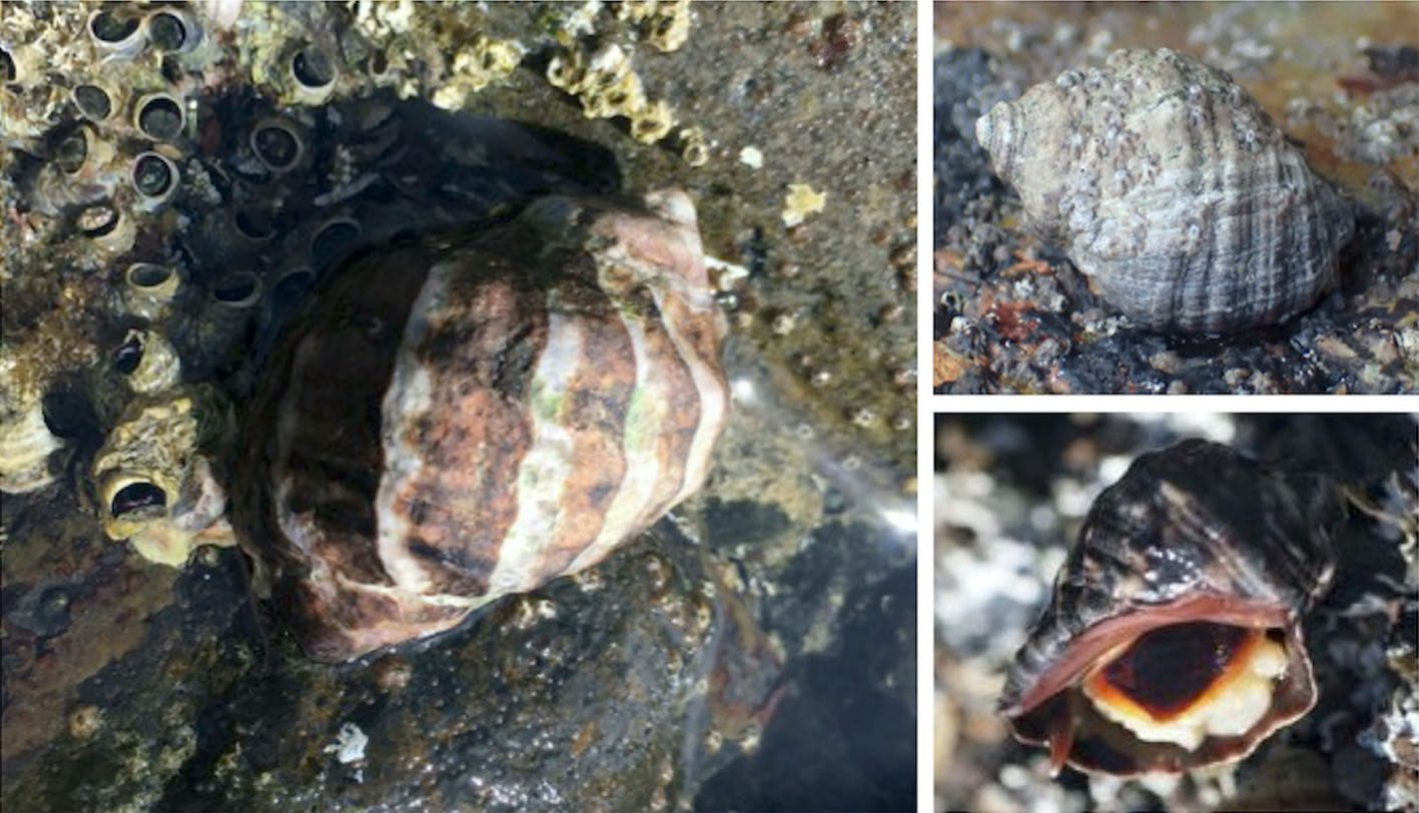
Color morphs of the range-shifting dark unicorn whelk *Mexacanthina lugubris.* Mexacanthina is distinguished from other intertidal whelks by its robust body, pronounced ridges and whorls, and the presences of a spine at the base of the aperture. Photographs by P. Wallingford

In the past few decades, the dark unicorn whelk, *Mexacanthina lugubris* (Fig. 1, hereafter referred to by genus), appears to have undergone a northern range shift into southern California, USA. Native to Baja California, Mexico, its range was recently reported as extending from Magdalena Bay, Baja California Sur to Laguna Beach, California (Marko and Vermeij 1999; Fenberg et al. 2014). The whelk was first reported in the San Diego area in 1974 (Radwin 1974) and was found at high abundances throughout San Diego by the 1990s (Hertz 1995), after which it continued to expand northward (Becker 2005). By the mid 2000s, *Mexacanthina* populations were reported more than 100 km north of San Diego at Thousand Steps Beach in Laguna Beach, CA, USA (Fenberg et al. 2014). One individual was documented at a site more than 50 km north of Thousand Steps, and a shell was observed at a site 80 km north of the established range limit (iNaturalist, Table 1). Peak abundances also appear to be shifting northward. From 2002 to 2014, densities as high as 36 individuals per m^2^ were found near Ensenada, Baja California, compared to only 2.4 per m^2^ at Cabrillo National Monument (Fenberg et al. 2014). It is unknown if *Mexacanthina* occurred in southern California prior to the 1970s: while there were no reports of live *Mexacanthina*, museum collections contain *Mexacanthina* shells that were collected in southern Orange County in 1937 and 1955 (Fenberg et al. 2014). The mechanisms of this recent expansion (or re-expansion) remain uncertain; like other whelks, *Mexacanthina* develop directly from benthic egg cases limiting the potential for dispersal via oceanic currents (Deng and Hazel 2010). Furthermore, rocky benches in southern California are often separated by significant expanses of sandy beaches. However, it is possible that spread could occur via egg cases attached to drifting algae, individuals moving subtidally, or human transport whether intentional or accidental.

**Table 1:** Survey sites and reports of *Mexacanthina* presence

Site (N–S)	Latitude	Longitude	Reported	Confirmed
Leo Carrillo Beach (MB)	34.044	− 118.937	iNaturalist 2017^a^	No
Point Fermin (PF)	33.705	− 118.295	No	No
Fisherman’s Cove (FC)	33.446	− 118.485	Pers. Comm. 2017	No
Little Corona (LC)	33.588	− 117.867	No	No
Crystal Cove (CC)	33.570	− 117.837	No	No
Crescent Bay (CS)	33.547	− 117.805	iNaturalist 2017	No
Shaw’s Cove (SH)	33.544	− 117.800	iNaturalist 2018	No
Heisler Park (HP)	33.543	− 117.793	MARINe 2013	Yes
Victoria Beach (VB)	33.520	− 117.765	iNaturalist 2019	Yes
Treasure Island (TI)	33.514	− 117.761	iNaturalist 2017	Yes
Table rock (TR)	33.502	− 117.747	Pers. Comm. 2018	Yes
**Thousand steps (TS)**	**33.493**	− **117.739**	**Yes**	**Yes**
Dana point (DP)	33.460	− 117.715	Yes	No
Carlsbad (CB)	33.132	− 117.337	Yes	Yes
Swami’s (SW)	33.035	− 117.294	Yes	Yes
Cardiff (CR)	33.000	− 117.279	Yes	Yes
Scripps (SC)	32.873	− 117.253	Yes	Yes
Wind and SEA (WS)	32.830	− 117.281	Yes	Yes
Sea ridge (SR)	32.808	− 117.267	Yes	Yes

For sites north of *Mexacanthina*’s published range limit (Thousand Steps, indicated in bold), the date and source of the first report are given. Shaded sites represent locations of abundance and density surveys, in addition to presence/absence surveys. Reported sightings are collated from published sources, monitoring programs, and community scientist observations. Sites are indicated as confirmed when *Mexacanthina* was found at least once during seasonal surveys

^a^Empty *Mexacanthina lugubris* shell

In coastal marine ecosystems, predatory mollusks are important intermediate predators that can shape community structure (Lubchenco and Menge 1978; Hughes and Burrows 1993; Navarrete 1996). As its range shifts north, *Mexacanthina* could affect communities by altering existing dynamics, such as through competition with native whelks. In southern California, the most common species are *Acanthinucella spirata* and *Nucella emarginata*. *Mexacanthina*’s current range extends farther south than those of southern California whelks, but there are large areas where multiple whelk species have historically co-existed. The southern range limit of *Acanthinucella* is Punta Baja, Baja California, approximately 700 km north of the southern range limit of *Mexacanthina* (Collins et al. 1996; Flagor and Bourdeau 2018), while the southernmost population of *Nucella* is reported to be at Punta San Thomas, Baja California, approximately 900 km north (Marko 1998). All three whelk species are generally found in mid intertidal zones along with their primary prey, the California mussel *Mytilus californianus* and acorn barnacles *Chthamalus fissus/dalli* and *Balanus glandula* (Connell 1970; Suchanek 1978; West 1986). *Mexacanthina* is reported to primarily feed on acorn barnacles (Marko and Vermeij 1999; Jarrett 2009) and potentially mussels (Becker 2005), similar to native whelks.

Competition is an important driver of range limits and community composition across spatial scales (Case et al. 2005), and ecologically and taxonomically similar species are most likely to interact strongly (Burns and Strauss 2011). If *Mexacanthina* competes with native whelks, native whelks may be at a disadvantage: *Mexacanthina* is typically larger and more robust, which could make the whelk better at foraging or less susceptible to predation (Hughes and Elner 1979; Thomas and Himmelman 1988). Additionally, *Mexacanthina* has a larger foot per surface area, which is beneficial for avoiding forceful removal by predators or waves (Rilov et al. 2004; Guerra-Varela et al. 2009). *Mexacanthina* is likely also better adapted to heat and desiccation stress due to their evolution in warmer locations. Average temperatures at its southern range limit are approximately 4 °C warmer than at the northern range limit (Table S1). Comparatively, temperatures at the southern range limits of native whelks are approximately 2 °C warmer (*Acanthinucella*) or roughly equivalent (*Nucella*). In the coming century, temperatures in southern California coastal communities are expected to increase by 1.8–5.5 °C (Cayan et al. 2008), resulting in a temperature regime more similar to that of Baja California Sur. If temperatures exceed native whelks’ physiological limits, *Mexacanthina* could become the dominant species. However, if niche-partitioning facilitates co-existence (Hutchinson 1959; Chase and Liebold 2003), southern California intertidal communities could ultimately resemble sites where these species have historically co-occurred.

To better understand the current impacts of *Mexacanthina* on southern California intertidal communities, as well as potential impacts associated with future climate change, we applied an integrative approach consisting of field observations and laboratory experiments. Our study addressed the following questions: (1) is *Mexacanthina* continuing to expand northward, (2) what are the potential impacts of *Mexacanthina* on native whelks, and (3) how might climate warming affect local and range-shifting whelks?

## Methods

To answer these questions, we reviewed historical and modern reports of *Mexacanthina* sightings and surveyed 20 intertidal sites, spanning approximately 250 km, for presence/absence of different whelk species to determine current ranges. At 10 of these sites, we surveyed whelk distributions and abundances to assess whether the presence and density of *Mexacanthina* was associated with altered distributions of native whelks. Potential impacts of *Mexacanthina* on native whelks were also assessed through a mesocosm feeding experiment, in which we manipulated densities of predators and species composition of both predators and prey. Finally, to predict how climate warming could affect different whelk species, we conducted thermotolerance trials to assess lethal temperature limits.

### Abundance and distribution

Evidence for *Mexacanthina* range shifts was collected from literature reviews, reports from biodiversity surveys conducted by MARINe, the Multi-Agency Rocky Intertidal Network, and PISCO, the Partnership for Interdisciplinary Studies of Coastal Oceans (https://marinedb.ucsc.edu/interactive/intertidalmap.html^2021^), as well as community science data (iNaturalist, https://www.inaturalist.org/taxa/292590-Mexacanthina-lugubris^2018^, communication with authors). Based on the reports of *Mexacanthina* sightings, we conducted presence/absence surveys at 20 sites in southern California, USA and abundance surveys across tidal elevations at 10 sites. Sites were chosen to represent a range of expansion history, with roughly equivalent division between regions where *Mexacanthina* is well-established (Thousand Steps to Sea Ridge), areas of reported/potential expansion (Crescent Bay to Table Rock), and areas where no expansion had been reported or expansion was unlikely (Leo Carrillo to Crystal Cove). Sites were selected across this range to assess differences in native whelk distributions between sites where *Mexacanthina* was present and absent.

Surveys were conducted quarterly beginning Fall 2017 through Summer 2018. Presence or absence of *Mexacanthina* and native whelks were determined using a 30-min timed count. During abundance surveys, we first laid a 25-m horizontal transect along the waterline and then laid five transects perpendicular to the horizontal transect every 5 m (starting at the 5-m mark). Vertical transects extended from the waterline to bare rock. Along each vertical transect, 1-m wide belts were surveyed, and the location of each whelk was recorded along the transect. Densities of whelks at each tide height (number of individuals/area in m^2^) were calculated from belt transect data using bins encompassing 0.25 m in vertical tidal elevation.

We used the distribution data to evaluate spatial overlap of *Mexacanthina* and native whelks. To account for zero-inflated and overdispersed data, we averaged tide height densities across transects at each site and used two-step gamma hurdle models, analyzing (1) native whelk presence/absence data based on a binomial distribution and (2) native whelk density data (> 0) based on a gamma distribution. We used separate models to compare the effects of *Mexacanthina* (presence or density), tidal elevation, and their interaction on native whelk distributions. Significance was evaluated via Wald Chi-square tests. We used R (R Core Team 2020) for all analyses.

### Species interactions

**Fig. 2:**
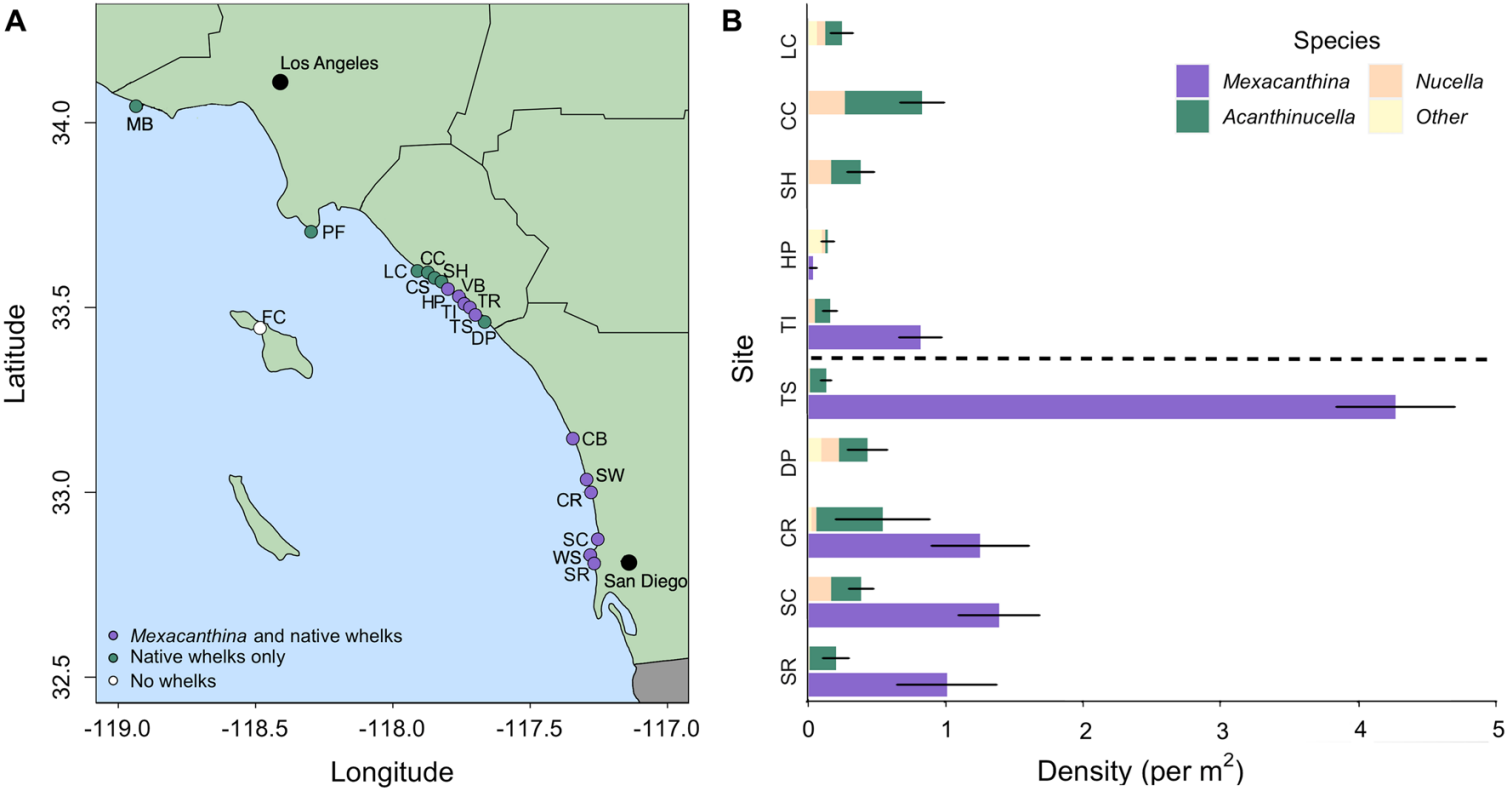
**A** *Mexacanthina* were found at 11 of the 20 sites surveyed (Table 1), including four sites north of its previously published range limit at Thousand Steps Beach (TS, indicated in bold). Native whelks were found at 19 of 20 sites. **B** Average densities (± SE) of *Mexacanthina* and native whelks at 10 sites where we conducted abundance surveys. Values are averaged across 5 transects and 4 seasons

To evaluate the potential for competition between *Mexacanthina* and native whelks, we manipulated species composition and density in a laboratory experiment using *Mexacanthina* and the most common native whelk *Acanthinucella* (referred to as M and A in treatments)*.* Whelks of both species were collected from Treasure Island Beach, Laguna Beach, CA, a site where *Mexacanthina* has recently expanded and where both species are abundant (Fig. 2). Individuals of similar sizes were collected to minimize differences in metabolic demand. Four tanks with programmed tidal cycles were used to simulate natural conditions experienced by the whelks and prey species, with a 12-h light period and low tide occurring twice daily for 2 h. Our experimental units were 11 by 11 cm mesocosms that contained 10 by 10 cm sandstone tiles.

Whelks were exposed to three prey treatments: mussels only, barnacles only, or a mix of both prey types. Whelks (up to 2 individuals per replicate, as described below) were continuously provided prey (either 4 mussels or 4 barnacle-covered shells) so that resources were not limited Mussels (32.76 ± 4.47 mm length) with and without barnacles were collected from Little Corona Beach in Newport Beach, CA. In the barnacle treatments, we bisected mussels and removed any soft tissue. We then counted the number of barnacles per half shell prior to attaching the shells to the tiles using silicone. In the mussel treatments, mussels were first scraped clean of epibionts. Using Vernier calipers, we measured mussel shell length (anterior to posterior), width (dorsal to ventral), and depth (left valve to right valve). Mussels were also attached horizontally to the tiles using silicone to ensure uniformity in surface area and distance between prey.

To quantify the strength of interactions and density-dependent effects of the whelks, we used six predator treatments consisting of one individual of each species (A, M), two individuals of each species (AA, MM), two individuals—one of each species (AM), and a control with no whelks (*C*; to account for any non-consumptive mortality across tanks). This was a randomized block design with 18 separate predator × prey treatments and a total of *n* = 4 replicates of each treatment (one per tidal tank, our blocking factor). Whelks were starved for one week prior to the experiment and were randomly assigned to treatments. Mussel mortality was assessed weekly, at which time mesocosm locations were shuffled within each tidal tank to minimize location effects. Barnacle mortality was assessed visually after four weeks (to ensure there were live prey remaining) and was quantified at the conclusion of the eight-week experiment.

Measurements of whelk shell and aperture length and width, as well as buoyant wet weight (Palmer 1982), were collected at the start and end of the experiment; dry weight was also determined at the end of the experiment. To compare biomass consumed between prey treatments, we created regression curves for biomass (ash free dry weight) to dimensions of mussels (*R*^2^ = 0.89, Fig. S1A) and biomass to number of barnacles (*R*^2^ = 0.83, Fig. S1B). Prey mortality in control tanks was not used in the analyses as no mussel mortality occurred within the control treatments and the average barnacle mortality accounted for less than 1% of mortality observed across predator treatments. Analysis of variance (ANOVA) was used to assess how prey (mussels, barnacles, or both) and predator composition (A, M, AA, MM, AM) affected biomass consumed and whelk growth (% change in mass). Biomass data were log-transformed to meet assumptions of normally distributed residuals. We did not include tank (our blocking factor) in the model as it did not significantly improve the model fit.

### Thermal tolerance

To determine the potential effects of warming, we estimated thermal tolerance of *Mexacanthina, Nucella,* and *Acanthinucella* by calculating each species’ LT_50_, or the temperature lethal to 50% of individuals. Whelks were collected from Treasure Island Beach, CA, and individuals (*n* = 5 per species per temperature) were placed in 1.5 mL centrifuge tubes with a piece of seawater-soaked chamois cloth (to prevent desiccation). We then randomly assigned whelks to one of six temperature treatments: 0 °C, 18.5 °C (ambient temperature), 32 °C, 35 °C, 38 °C, and 41 °C. Temperature treatments were chosen based on preliminary experiments and previously collected long-term environmental data in Laguna Beach, CA (Wallingford and Sorte 2019; Pandori and Sorte 2021). These temperatures represent the gradient of maximum temperatures experienced across *Mexacanthina*’s range (Table S1) and include the full range of outcomes for all species (from 0 to 100% survival) to quantify LT_50_ (50% survival). We also conducted mortality assessments at 0 °C to assess whether whelk survival was limited by cold temperatures.

Using 28-L digital water baths, tubes were heated from ambient to treatment temperatures over a 40-min period (Sorte et al. 2019). Following a 6-h exposure period, whelks were transferred to a recirculating seawater system for an 18-h recovery period, at which time mortality was assessed. We used logistic regressions to calculate LT_50_, as well as differences in survival between species and treatments.

## Results

### Northward expansion

**Fig. 3:**
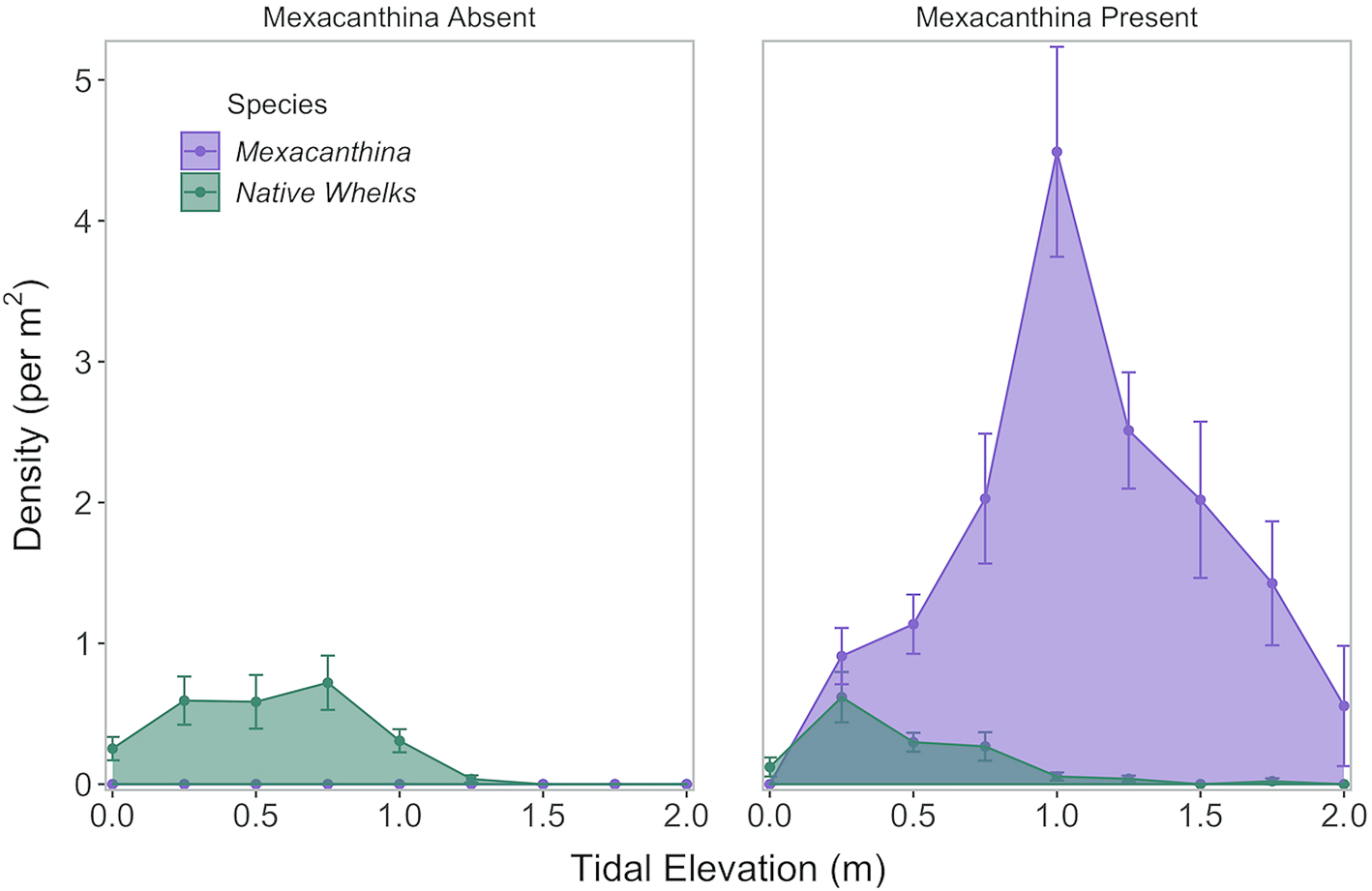
Average densities (± SE) of *Mexacanthina* and native whelks across tide heights. Values are averaged across sites and seasons (*n* = 40 total surveys) and are separated by sites where *Mexacanthina* is present (*n* = 6) versus absent (*n* = 4)

During presence/absence surveys, *Mexacanthina* was found at 11 of the 20 sites we surveyed, including 4 sites north of its previously documented range (Fig. 2). Native whelks were found at 19 of the 20 sites. Across sites, average *Mexacanthina* densities (where present) ranged from between 0.05 and 4.27 individuals per m^2^ (Fig. 2). During our surveys, we found the highest densities of *Mexacanthina* at Thousand Steps (maximum of 24.55 individuals per m^2^). Average native whelk densities ranged from 0.14 to 0.84 individuals per m^2^. *Mexacanthina* and native whelks also showed different distribution patterns, with *Mexacanthina* found at higher tidal elevations more often than native whelks (Fig. 3).

### Potential impacts

**Fig. 4:**
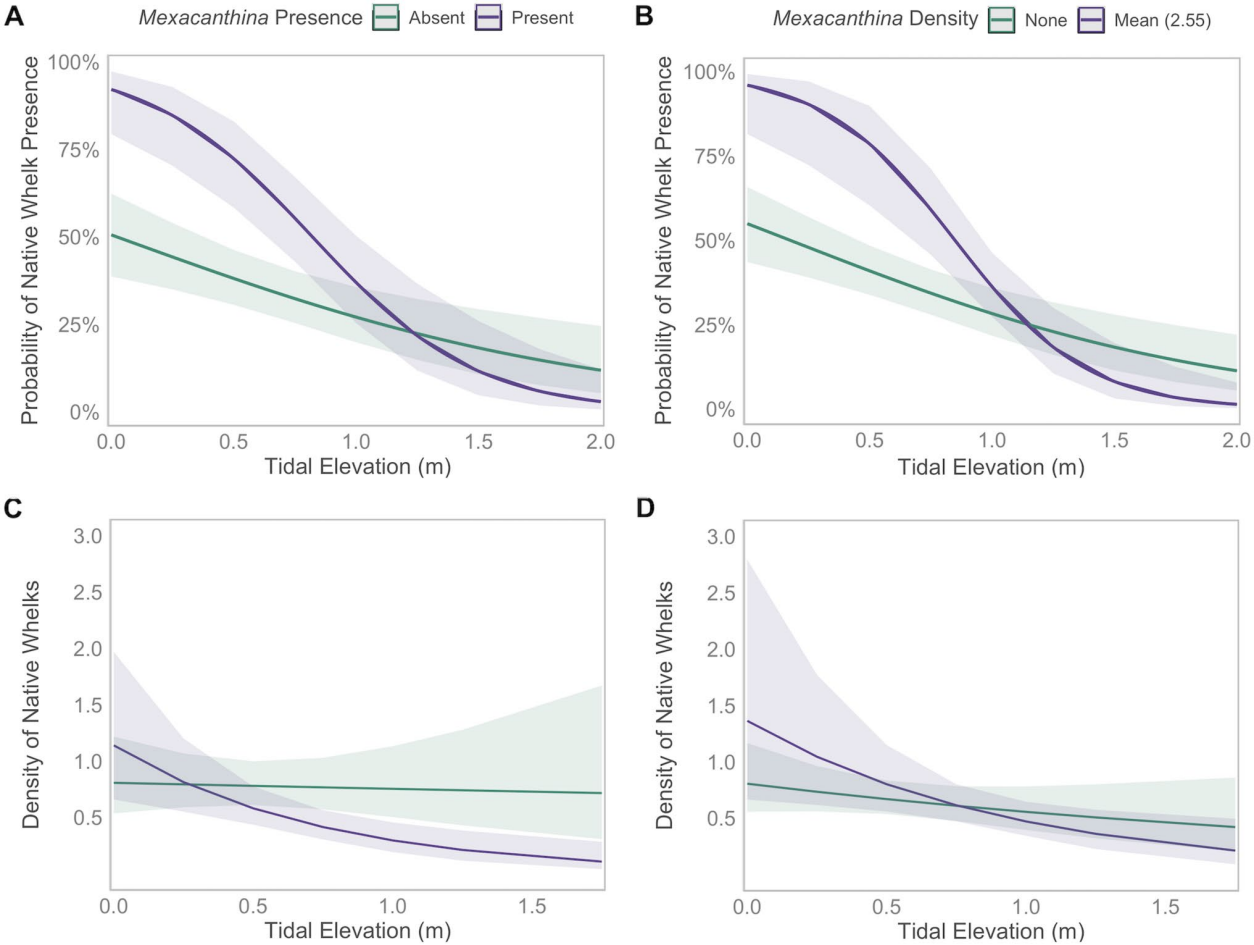
Native whelks occurred less often at higher tide heights when **A** *Mexacanthina* was present and **B** as *Mexacanthina* density increased (binomial distributions). Native whelk density (> 0) also decreased across tidal elevations with **C** *Mexacanthina* presence and **D** *Mexacanthina* density (Gamma distributions). Panels show responses (± SE) of native whelks across transects, sites, and seasons (*n* = 200)

Hurdle (two-step) models were used to evaluate the effects of *Mexacanthina*, tidal elevation, and their interaction on native whelk (1) presence and (2) density. For both models (*Mexacanthina* presence versus *Mexacanthina* density), we found a positive association between *Mexacanthina* and native whelk presence, which was driven by spatial overlap at low elevations. Binomial regressions showed that the probability of native whelks being present increased significantly when *Mexacanthina* was present (*χ*^2^ = 18.77, *p* < 0.001; Table S2, Fig. 4) and as *Mexacanthina* density increased (*χ*^2^ = 17.42, *p* < 0.001). *Mexacanthina* was found higher in the intertidal than native whelks (Fig. 3) while native whelks were found less often as tidal elevation increased (*χ*^2^ = 12.93, *p* < 0.001; *χ*^2^ = 18.63, *p* < 0.001). Native whelk presence was also impacted by the interactions between tidal elevation and *Mexacanthina* presence (*χ*^2^ = 9.49, *p* = 0.002) and density (*χ*^2^ = 13.87, *p* < 0.001): when *Mexacanthina* was present and at high densities, native whelk occurrences increased at low tide heights but were less likely higher on the shore.

When native whelks were present, densities were similarly associated with *Mexacanthina*: there was a positive association between densities of native whelks and *Mexacanthina* at low tidal elevations, and a negative association at high elevations. Gamma generalized linear models showed a significant interactive effect with native whelk densities decreasing at higher elevations when *Mexacanthina* was present (*χ*^2^ = 5.34, *p* = 0.02) and as *Mexacanthina* densities increased (*χ*^2^ = 5.34, *p* = 0.04). Native whelk densities did not change with the main effects of *Mexacanthina* presence (*χ*^2^ = 0.96, *p* = 0.32) or density (*χ*^2^ = 2.92, *p* = 0.09). Tidal elevation also had no main effect (*χ*^2^ = 0.03, *p* = 0.86; *χ*^2^ = 1.21, *p* = 0.27).

**Fig. 5:**
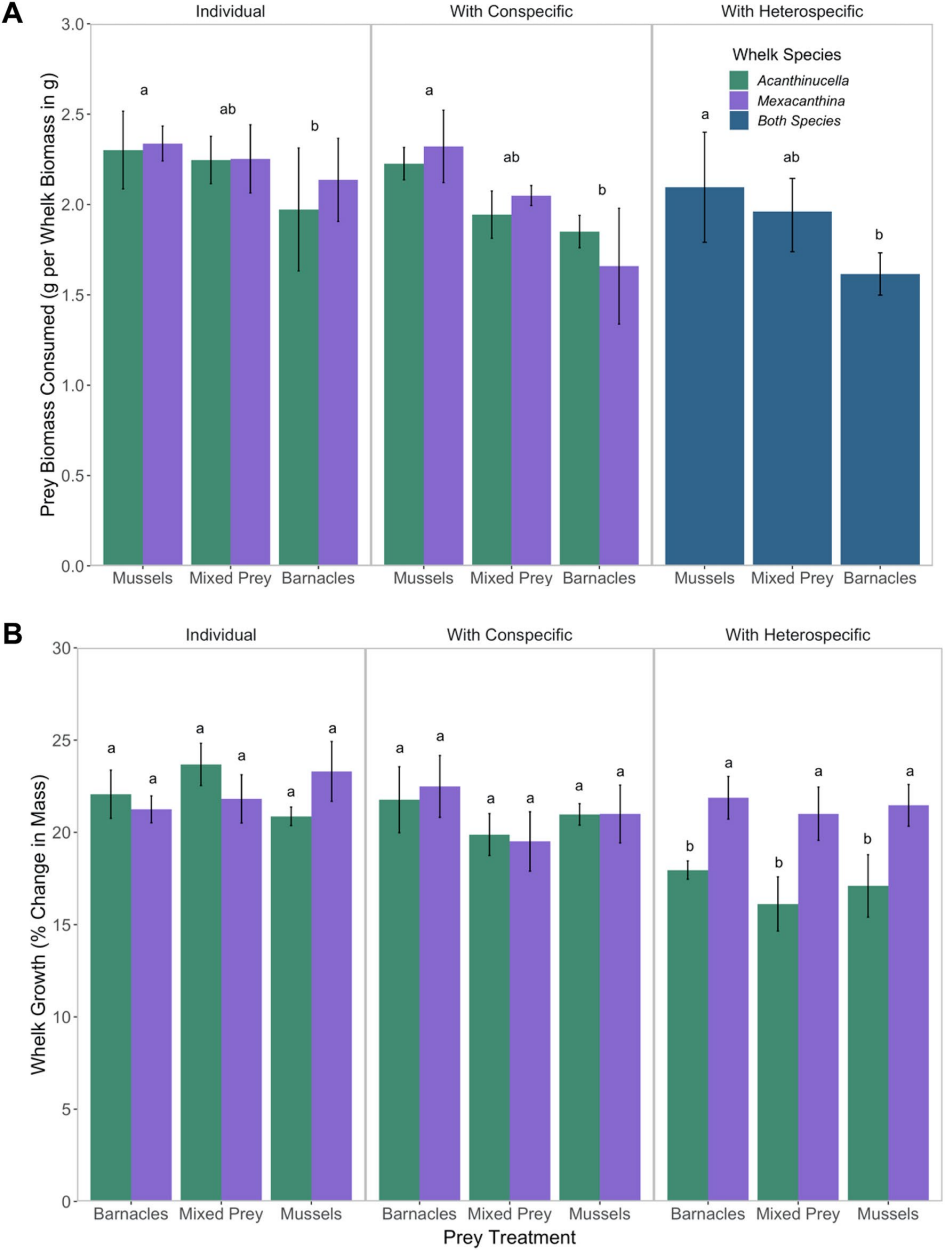
**A** Average biomass of prey consumed (g) per whelk biomass (g) varied across prey treatments, with less biomass consumed in the barnacle only treatments compared to mussel only treatments. **B** *Acanthinucella* experienced reduced growth (% change in mass) in mixed-predator treatments. Values are averages (± SE) of *n* = 4 replicates

In the competition experiment, biomass of prey consumed differed across prey treatments (*F* = 5.21, *p* = 0.009, Table S3), but there was no effect of predator treatment (*F* = 1.47, *p* = 0.23) or the interaction (*F* = 0.24, *p* = 0.98; Fig. 5). Both *Mexacanthina* and native *Acanthinucella* consumed more biomass in treatments containing only mussels compared to those containing only barnacles (Fig. 5). Whelk growth (% change in mass) did not differ across prey treatments (*F* = 4.83, *p* = 0.50), but there was a significant difference between predator treatments (*F* = 6.38, *p* < 0.001), with *Acanthinucella* growing less in the mixed whelk treatment than when alone or with a conspecific (AM vs. A and AA treatments). There was not a significant interaction between predator and prey treatments on growth (*F* = 4.85, *p* = 0.71).

**Fig. 6:**
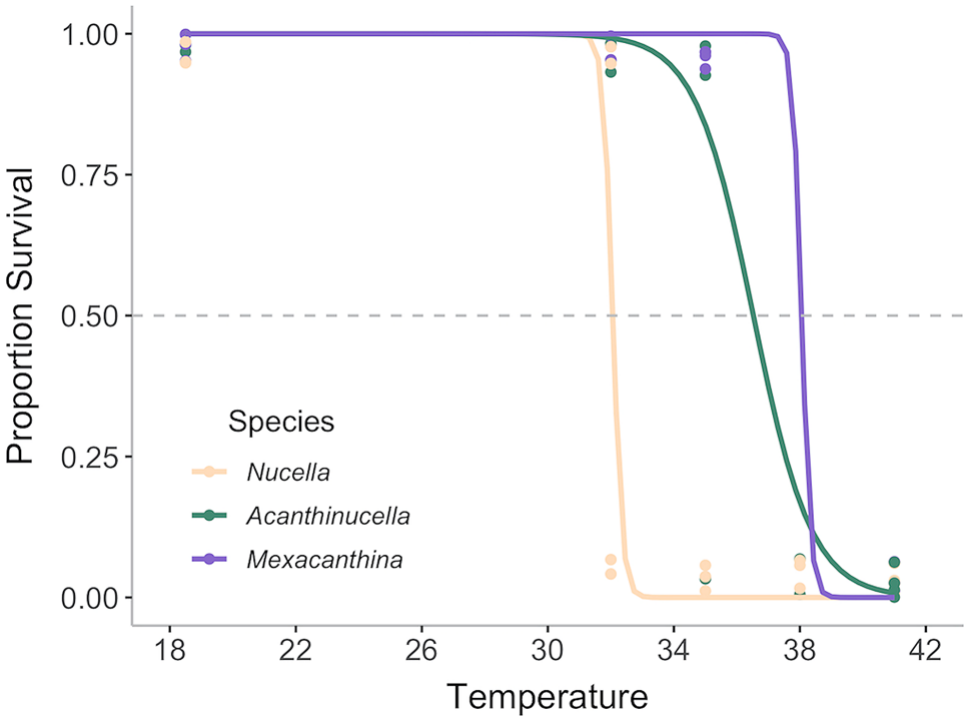
Logistic regression showing proportional survival following a thermotolerance assays. *Mexacanthina* and *Acanthinucella* were more heat tolerant than *Nucella*, with calculated LT_50_ values of 38.1 °C, 36.5 °C*,* and 32.1 °C, respectively. Data points represent survival of individual replicates at each temperature treatment (*n* = 5) and are jittered for visibility

We conducted thermotolerance assays to explore the potential for future climate warming to influence whelk survival, which could influence population sizes and species interactions. We calculated LT_50_ values of 38.1 °C for *Mexacanthina*, 36.5 °C for *Acanthinucella,* and 32.1 °C for *Nucella* (Table S4)*.* There was a significant difference in survival between temperature treatments (*χ*^2^ = 90.80, *p* < 0.001; Fig. 6) and species (*χ*^2^ = 27.45, *p* < 0.001). A Tukey post hoc test showed no differences in response between *Mexacanthina* and *Acanthinucella* (*z* = 1.61, *p* = 0.22), but both whelk species were significantly different than *Nucella* (*z* = 2.99, *p* = 0.007 and *z* = 2.66, *p* = 0.02, respectively).

## Discussion

In southern California, we found that *Mexacanthina* is continuing to expand northward and that the range-shifting whelk can impact native whelks. *Mexacanthina* uses similar resources and habitats as native whelks, and densities can be an order of magnitude higher. *Mexacanthina* is also able to use space at higher tidal elevations, and native whelks occur less often in the high intertidal at sites where *Mexacanthina* is present. In a mesocosm experiment, we observed that the native whelk *Acanthinucella* grew less in treatments that contained *Mexacanthina,* regardless of prey type*.* As climate change continues, environmental conditions will likely become more stressful for all species. However, *Mexacanthina* populations are likely to be at an advantage relative to native species, as they have higher thermal tolerances and already occupy warmer habitats than native whelks.

Over the course of a year, we confirmed the presence of *Mexacanthina* at four sites up to 5 km north of its previously documented range edge at Thousand Steps. However, we were unable to confirm the presence of *Mexacanthina* at four other sites where it had been previously reported. Our surveys do not preclude the presence of *Mexacanthina* at these locations, as low densities might limit observations. The challenge of confirming *Mexacanthina*’s presence at new sites raises an important point about tracking range-shifting species. Historical ranges for many species are unknown or imprecise, and often can vary spatially and temporally (Pereyra 2020). Without ongoing monitoring, it can be difficult to detect when exactly a range-shifting species enters a community. While we confirmed some reports of *Mexacanthina* beyond its documented range limit*,* more frequent sampling would be needed to determine when *Mexacanthina* expands to a specific site. Our study relied on data from ongoing monitoring programs, as well as reports from community scientists. While search efforts are often not reported in these instances, visitors to tidepools were almost always the first to report *Mexacanthina* at a new site. When tracking range shifts, community scientists can increase sampling coverage and offer valuable insights that help fill gaps in our knowledge of species distributions (Soroye et al. 2018; Pecl et al. 2019).

Reports of observations at new sites suggest that dispersal is ongoing. During our surveys, occurrences varied across seasons at Heisler Park, the northernmost site where we observed *Mexacanthina*. However, high densities at other sites, most notably Treasure Island and Thousand Steps, suggest that as populations at the range edge become well-established, increased and more consistent propagule pressure could lead to greater numbers of individuals at and beyond the current range limit (Gaines et al. 2007). Evidence for climate-driven range shifts often also includes a range contraction at the range edge that is becoming more physically stressful (Sheth and Angert 2018). Decreasing *Mexacanthina* populations in southern Baja California, Mexico support this assessment. Museum collections indicate a northward shift in the southern range edge, previously reported to be Magdalena Bay, Baja California Sur. Surveys conducted in 2014 found no *Mexacanthina* in Baja California Sur south of 26.05° N, although specimens were collected in 1950–1979 from areas between 23.9 and 24.8° N (Fenberg et al. 2014). The northward expansion of *Mexacanthina* may be due to greater environmental stress at more southern locations.

As *Mexacanthina* shifts north, it has the potential to disrupt existing community dynamics through interactions with local species. *Mexacanthina* uses similar habitats and resources as native whelks, primarily preying on the California mussel *M. californianus* and the acorn barnacles *Chthamalus dalli/fissus* and *B. glandula* (West 1986; Deng and Hazel 2010)*.* Native whelks were more likely to be present, and at higher densities, at sites where *Mexacanthina* were present. This is likely due to greater prey availability, specifically mussels, at these sites, representing a bottom-up effect acting on both native whelks and *Mexacanthina.* This is supported by the comparable abundances of native whelks at Shaw’s Cove and Crystal Cove, where *Mexacanthina* is absent*.* Although they share habitat space and prey, in most locations, prey availability is currently unlikely to be a limiting resource. However, if prey species continue to decline (e.g., Smith et al. 2006), then the potential for competitive impacts could increase.

Despite the low probability of direct negative effects via interference competition at the site level (due to high prey availability), our results suggest that there could be negative trait-mediated impacts of *Mexacanthina* on native whelks as abiotic stress increases. At sites with *Mexacanthina* and when *Mexacanthina* densities were high*,* native whelk presence and densities were negatively associated with *Mexacanthina* at higher tidal elevations. This suggests that niche-partitioning may be occurring, with elevational distributions of natives shifting downward in the presence of the range-shifting species. For intertidal whelks, abiotic and biotic stress typically determine distributions, with desiccation and temperature increasing in the high intertidal compared to greater predation in the low intertidal (Paine 1969; Menge and Sutherland 1976; Rilov and Schiel 2006). As temperatures increase, populations living at or near their thermal limits will experience declines leading to range contractions (Wallingford and Sorte 2019; Sorte et al. 2019). Additionally, metabolic demands increase with warming, requiring greater amounts of time spent foraging to meet the same energetic demands and exposing whelks to greater risk from both biotic and abiotic factors (Sanford 2002). Because of its greater thermal tolerance, *Mexacanthina* could access more prey and habitats than native whelks and spend longer amounts of time at tidal elevations where the species overlap. Future studies could examine how distributions differ in areas where species have historically co-existed to determine if length of interactions or a shared evolutionary history alter the patterns we observed.

Another potential mechanism that could explain the negative association between *Mexacanthina* and native whelks at higher tidal elevations is intraguild predation, in which predators consume species at their same trophic level. Previous studies have reported acorn barnacles as the primary prey of *Mexacanthina* (Marko and Vermeij 1999; Jarrett 2009; although see Becker 2005). However, we found that the whelks consumed greater numbers of mussels than barnacles in lab conditions and appeared to consume a diverse set of prey items in the field, including herbivorous gastropods and other whelks (Wallingford pers. obs.). In addition to being consistent with our survey results (i.e., the negative association at high tidal elevations), intraguild predation could explain native whelks’ reduced growth under lab conditions if *Acanthinucella* individuals are less likely to forage when *Mexacanthina* is present (Holt and Polis 1997). Previous studies have shown that intraguild predation affects whelk behavior and physiology: when sea stars are present, *Nucella* forage less often, experiencing reduced growth and reproductive ability (Gosnell et al. 2012), and even undergo shifts in diet (Sanford et al. 2003). While no predation between whelks occurred during lab experiments, future field experiments could help elucidate the relationship between the whelks, both as competitors and potentially as intraguild prey.

An important caveat is that *Mexacanthina* overlaps with *Acanthinucella* and *Nucella* across much of its range, and it is unclear the degree to which current distributions in areas of recent *Mexacanthina* expansion are influenced by that evolutionary history. In our mesocosm experiment, we specifically looked at how *Acanthinucella* individuals interacted with *Mexacanthina* at a site where the latter had recently expanded. We did not test how these relationships might differ at sites where both species are endemic or at sites where *Mexacanthina* has not established. Given their history, native whelks might recognize *Mexacanthina* as a threat, which could explain the differences we observed in distribution patterns. This might also account for the lower growth rate we observed for *Acanthinucella* in the mesocosm experiment if native whelks avoid *Mexacanthina*, and forage less, when they are in close proximity. Sites where *Mexacanthina* has recently established may ultimately resemble sites where the species have historically co-existed, although it is difficult to estimate the time frame for such shifts in community composition. If *Mexacanthina* continues to shift north, further research on how these sites are changing, incorporating data on the abundances and distributions of native whelks before and after the arrival of *Mexacanthina*, could provide a more complete picture of these dynamics.

Interestingly, the native whelk *Acanthinucella* has also recently undergone a northern range shift along the California coast in response to climate change (Hellberg et al. 2001; Flagor and Bourdeau 2018). This range shift shares a number of similarities with that of *Mexacanthina,* with new populations found 2° of latitude north of the previously documented range limit, despite limited dispersal potential due to direct-developing young (Flagor and Bourdeau 2018). While many, if not most, species are likely to undergo range shifts under changing climatic conditions, community fragmentation could occur if species shift asynchronously, as appears to be the case with the whelk guild presented here. In some cases, range-shifting species may compete with natives that are not able to undergo range shifts (or are unable to shift on pace with climate change), potentially leading to local extinctions. If endemic species become locally extinct, range-shifting species may be able to fill a comparable niche, but there are likely to be long-term effects, such as a shift in species assemblages and changes to population dynamics of interacting species (Flagor and Bourdeau 2018; Aguilera et al. 2020). Because communities are unlikely to shift as a whole, climate-driven range shifts have the potential to alter community composition and ecosystem functioning. Climate change will dramatically alter existing ecological communities, necessitating a broader view of which species constitute a native community (Wallingford et al. 2020).

When climate-driven range shifts occur, they have the potential to alter existing communities through changes to species interactions. In this study, we found that the dark unicorn whelk *Mexacanthina lugubris* is undergoing a northern range expansion, with native whelks displaying altered distributions in the field and changes to energy allocation in the lab when the range-shifter is present. Furthermore, native whelks are likely to experience greater impacts as climate change continues and accelerates, due to greater vulnerability to abiotic stress. Although species composition of this intermediate predator guild is likely to change in the future, ecosystem functioning might be maintained through functional redundancy as *Mexacanthina* appear to fill a similar niche as native whelks. Range shifts present an opportunity for individual species and ecosystem services to persist in the face of climate change, although future communities may be different than those we recognize today. Understanding and monitoring how communities respond is increasingly important, as range shifts can impact local communities while also being vital for preserving global biodiversity.

## Supplementary Information

Below is the link to the electronic supplementary material.

Supplementary file1 (PDF 571 KB)

## Declarations

### Data

Data and analyses are available at https://github.com/piperw/darkunicorn.
