# Supplementary material for: Dynamic species interactions associated with the range-shifting marine gastropod Mexacanthina lugubris
Source: Oecologia. 2022 Mar 7;198(3):749–61. doi: 10.1007/s00442-022-05128-5 (PMC8956515; doi:10.1007/s00442-022-05128-5)

Electronic Supplementary Material: Dynamic species interactions associated with the range-shifting marine gastropod *Mexacanthina lugubris*

Piper D. Wallingford*[1,2] and Cascade J. B. Sorte[1]

[1]Department of Ecology and Evolutionary Biology, University of California, Irvine, CA

[2]Department of Ecology and Evolutionary Biology, University of California, Los Angeles, CA

* Corresponding Author ORCID iD:0000-0003-2385-9590 email: pwallingford@ucla.edu

Table S1 Historical mean, mean daily maximum, and maximum temperatures for sites across *Mexacanthina*'s range and our survey area, from south to north. Data were obtained from the Servicio Meteorológico Nacional (Mexico) and the National Weather Service (USA)

Site	Latitude	Longitude	Mean	Mean Max	Max	Period
Puerto San Carlos	24.79	-112.11	20.9	36.0	42.5	1981-2010
San Juanico	26.26	-112.48	19.7	37.5	42.0	1981-2010
Bahia Tortugas	27.69	-114.90	19.7	36.0	43.0	1981-2010
Santa Rosalita	28.67	-114.24	18.5	42.5	47.0	1981-2010
Ensenada	31.86	-116.61	17.1	34.7	39.0	1981-2010
San Diego	32.87	-117.25	18.1	35.0	41.7	1980-2018
Oceanside	33.12	-117.38	16.2	35.6	41.1	2000-2018
Laguna Beach	33.54	-117.81	16.9	33.9	38.9	1980-2018*
Newport Beach	33.62	-117.94	16.9	31.1	35.0	1980-2018
Santa Monica	34.01	-118.50	16.3	30.6	37.2	1980-2013

* Mean weather data available 1980-2010

Table S2 Analysis of deviance tables for Gamma hurdle models (Wald chisquare tests). We analyzed the effects of *Mexacanthina* presence and density, tidal height, and their interaction on native whelk presence (zero and non-zero data based on a binomial distribution) and densities (non-zero data based on a gamma distribution)

Gamma Hurdle Model - *Mexacanthina* Presence

		Binomial Distribution		Gamma Distribution	
Fixed Effects	dF	χ^2	P	χ^2	P
Mexacanthina	1	18.77	< 0.001	0.96	0.32
Tide Height	1	12.93	< 0.001	0.03	0.86
Interaction	1	9.49	0.002	5.32	0.02

Gamma Hurdle Model - *Mexacanthina* Density

		Binomial Distribution		Gamma Distribution	
Fixed Effects	dF	χ^2	P	χ^2	P
Mexacanthina	1	17.42	< 0.001	2.92	0.09
Elevation	1	18.63	< 0.001	1.21	0.27
Interaction	1	13.87	< 0.001	5.34	0.04

Table S3 Summary of two-way ANOVA results of the mesocosm experiment. We analyzed the effects of predator and prey treatments on biomass consumed(g/whelk g) and growth (% change in mass)

	Biomass			Growth		
Parameter	df	F	P	df	F	P
Predator	4	1.47	0.23	5	6.38	< 0.001
Prey	2	5.21	0.009	2	4.83	0.50
Predator x Prey	8	0.24	0.98	10	4.85	0.71
Residuals	45			54		

Table S4 Analysis of deviance tables for generalized linear models and results of Tukey Post-Hoc comparisons of thermotolerance assays. We compared the effects of species and temperature treatment on whelk survival, using a binomial distribution

Binomial Regression Results

Parameter	df	χ^2	P
Species	2	27.45	< 0.001
Temperature	1	90.80	< 0.001

Tukey Post-Hoc Results

Species Comparison	z	P
Acanthinucella - Nucella	2.66	0.02
Mexacanthina - Nucella	2.99	0.007
Mexacanthina - Acanthinucella	1.61	0.22

Fig. S1 Biomass regressions for (a) mussels, based on length x width ($R^2 = 0.89$), and (b) barnacles, based on counts of individuals ($R^2 = 0.83$)

A

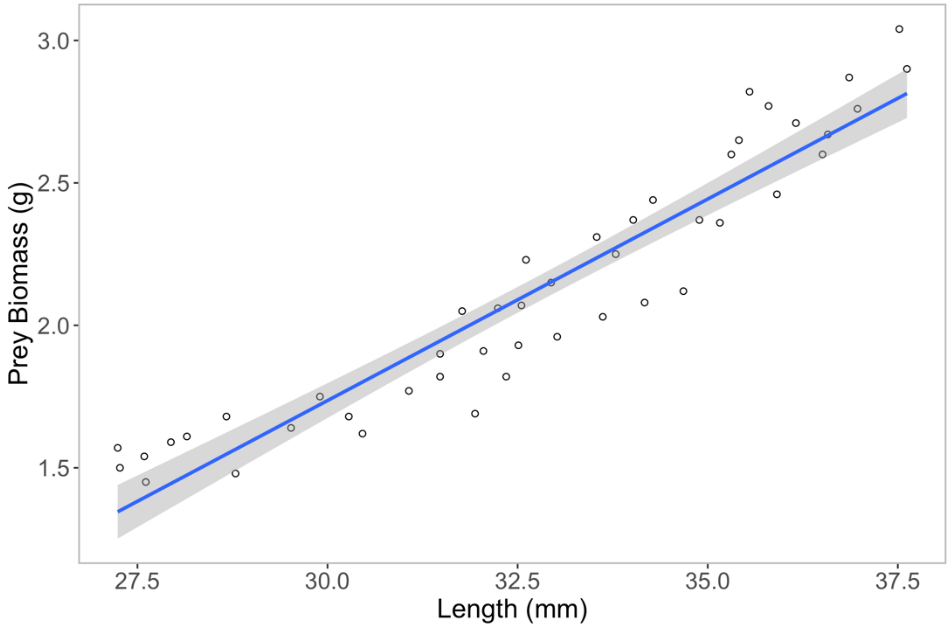

B

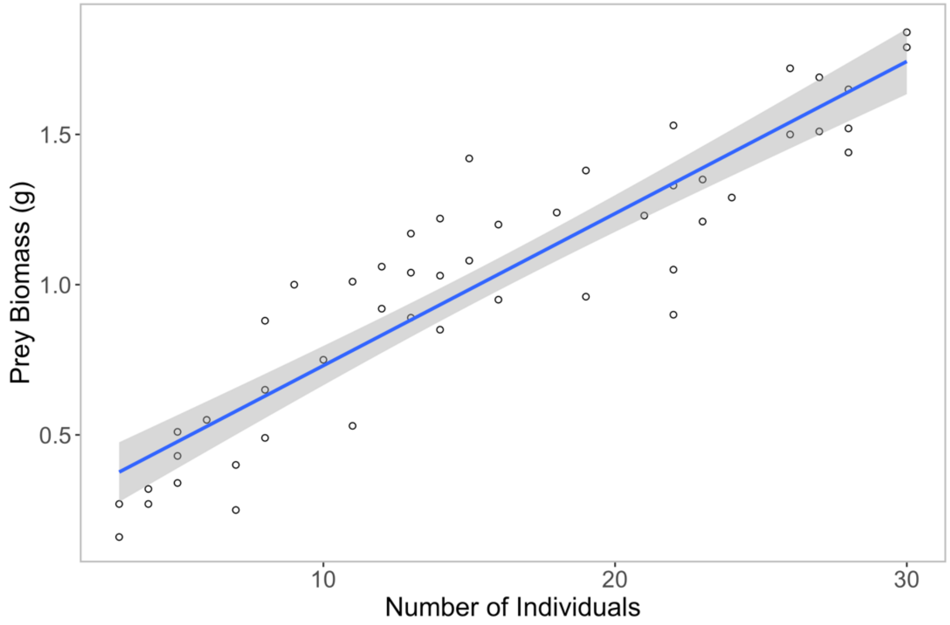

Fig. S2 Native whelk A) presence and B) densities at different *Mexacanthina* densities: 0, minimum, mean, and maximum. Figures show responses of native whelks across transects, sites, and seasons (n = 200) ± SE

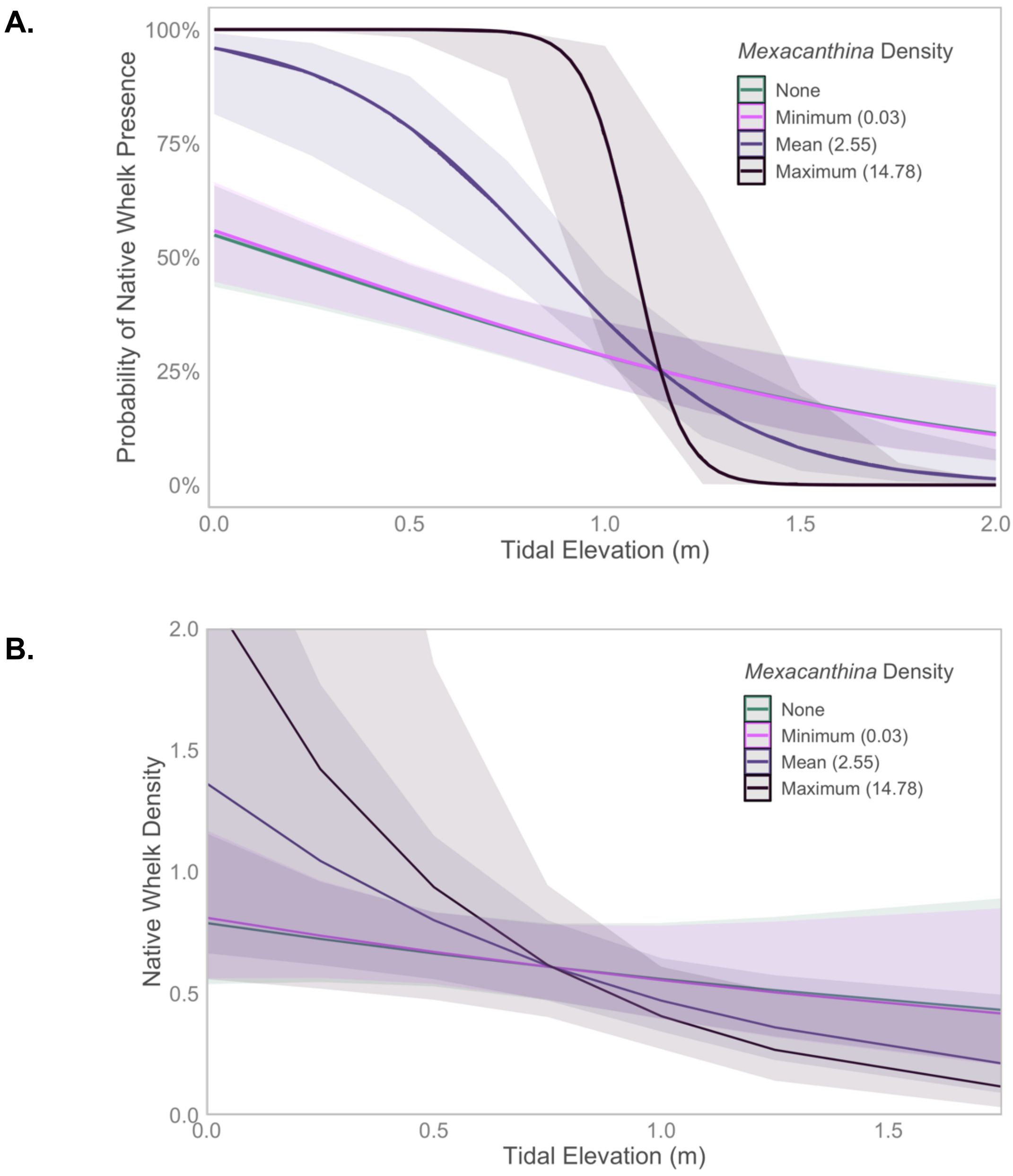

Supplement: Supplementary file 1 — Supplementary file1 (PDF 571 KB) [file 442_2022_5128_MOESM1_ESM.pdf]
